# Prevalence, Virulence Gene Distribution and Alarming the Multidrug Resistance of *Aeromonas hydrophila* Associated with Disease Outbreaks in Freshwater Aquaculture

**DOI:** 10.3390/antibiotics10050532

**Published:** 2021-05-04

**Authors:** Doan Thi Nhinh, Dung Viet Le, Kim Van Van, Nguyen Thi Huong Giang, Lua Thi Dang, Truong Dinh Hoai

**Affiliations:** 1 Faculty of Fisheries, Vietnam National University of Agriculture, Hanoi 131004, Vietnam; dtnhinh@vnua.edu.vn (D.T.N.); levietdung@vnua.edu.vn (D.V.L.); kvvan@vnua.edu.vn (K.V.V.); 2 Faculty of Veterinary Medicine, Vietnam National University of Agriculture, Hanoi 131004, Vietnam; nthuonggiangtycd@vnua.edu.vn; 3 Research Institute for Aquaculture No 1, Bac Ninh 16352, Vietnam; danglua@ria1.org; 4 Faculty of Agriculture, University of Miyazaki, Miyazaki 889-2192, Japan

**Keywords:** *Aeromonas hydrophila*, infection prevalence, virulence genes, antimicrobial resistances, freshwater fish, Vietnam

## Abstract

The study aims to evaluate the infection prevalence, virulence gene distribution and antimicrobial resistance of *Aeromonas hydrophila* associated in diseased outbreaks of cultured freshwater fish in Northern Vietnam. The confirmed *A. hydrophila* were screened for the presence of the five pitutative-virulence genes including aerolysin (*aerA*), hemolysin (*hlyA*), cytotonic enterotoxin (*act*), heat-labile cytotonic enterotoxin (*alt*), and heat-stable enterotoxin (*ast*), and examined the susceptibility to 16 antibiotics. A total of 236 *A. hydrophila* isolates were recovered and confirmed from 506 diseased fish by phenotypic tests, PCR assays, and *gyrB*, *rpoB* sequenced analyses, corresponding to the infection prevalence at 46.4%. A total of 88.9% of *A. hydrophila* isolates harbored at least one of the tested virulence genes. The genes *aerA* and *act* were most frequently found (80.5% and 80.1%, respectively) while the *ast* gene was absent in all isolates. The resistance to oxacillin, amoxicillin and vancomycin exhibited the highest frequencies (>70%), followed by erythromycin, oxytetracycline, florfenicol, and sulfamethoxazole/trimethoprim (9.3–47.2%). The multiple antibiotic resistance (MAR) index ranged between 0.13–0.88 with 74.7% of the isolates having MAR values higher than 0.2. The results present a warning for aquaculture farmers and managers in preventing the spread of *A. hydrophila* and minimizing antibiotic resistance of this pathogen in fish farming systems.

## 1. Introduction

Freshwater fish farming represents a substantial proportion in the aquaculture industry, particularly in developing countries providing food and livelihood and contribution to the regional economy [1]. A great diversity of freshwater aquatic organisms has been cultured, of which, tilapia; carp; and catfish are the major species in global aquaculture production [1,2]. Recently, the farming systems of these species have been shifted from simple traditional to intensive culture methods [1,3]. In the intensive practices, animals are confined in densely stocked and often high organic load conditions which trigger the development and spread of pathogenic bacteria [4,5]. The problems and economic losses related to bacterial diseases have, therefore, become increasingly frequent.

*Aeromonas* spp. are commonly found in freshwater, estuarine, and saltwater environments [6,7,8]. Members of this genus have been the focus of attention because of their potential to act as pathogens on a wide range of hosts not only in fish, amphibians, and reptiles but also in mammals, including humans [8,9]. Among *Aeromonas* species that cause fish diseases, *A. hydrophila* has been identified as one of the most dangerous pathogens causing mortality outbreaks in a diversity of cultured fish species [6,10] and has been responsible for huge economic losses in various countries [11,12].

The infection of farmed and wild fish by *A. hydrophila* is characterized as hemorrhagic septicemia with signs of ulceration, hemorrhaging, and fin erosion [6]. The disease displays chronic traits that persist for weeks, the mortality rate gradually increases and high cumulative mortality can occur [13]. More critically, due to the capacity of human infection, the *A. hydrophila* diseased fish proposed a potential risk to human health through foodborne [14]. The capacity of *A. hydrophila* to cause diseases relates to a variety of virulence factors which are complex and multifactorial [8,15]. Among them, hemolysin (*hlyA*), aerolysin (*aerA*), cytotoxic heat-labile enterotoxin (*act*), cytotonic heat-labile enterotoxin *(alt)*, and cytotonic heat-stable enterotoxin (*ast*) are well-known virulence factors and likely related to the hemorrhagic septicemia symptoms on the infected fish and animals [16,17].

To prevent and treat bacterial infections in aquaculture, antibiotics are commonly used via medicated feed or direct addition to the culture water [18]. These administration methods often result in heavy use of antibiotics, broader aquatic areas exposed and a wide range of bacteria impacted to the drugs in comparison to the use of antibiotics in terrestrial animal production [19]. The potential consequences of antibiotic overuse or misuse include the development of antibiotic-resistant bacteria, transferring of resistance traits to the bacterial community, and reducing the efficacy of antibiotic treatment for human and animal diseases [20,21].

Tilapia, channel catfish and carp (common carp and grass carp) are main culture species in Northern Vietnam. Their fast growth rate, ease to culture, and ability to fetch very good market prices have contributed towards the rapid expansion of the culture area, and they have become favorable culture species in this region. Although intensive farming systems of those freshwater fish have rapidly developed, increased in economic importance, and contributed to the achievement of the top 3 seafood exporting countries [22], the information on infection by *A. hydrophila* in freshwater fish is limited, particularly in Northern Vietnam. The aim of this study was, therefore, to identify the prevalence of infection, the presence of the genes encoding relevant virulence factors and antimicrobial resistance of *A. hydrophila* isolated from freshwater fish in this region.

## 2. Results

### 2.1. Clinical Signs and Gross Lesions

**Figure 1:**
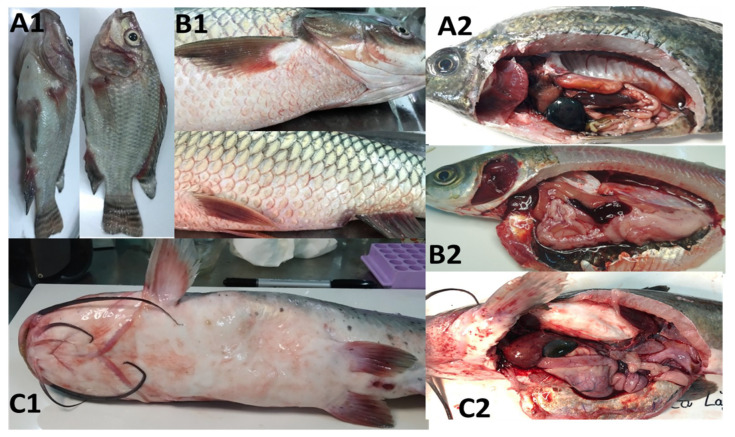
Clinical symptoms and gross lesions of diseased fish infected with *A. hydrophila*. The clinical signs of heavy hemorrhage around the fish’s mouth, operculum, and fin bases, and fin erosion of tilapia (**A1**), grass carp (**B1**) and channel catfish (**C1**); and gross lesions of enlarged gall bladder and liver, enlarged and darkened spleen, hemorrhage and empty stomach and intestines in infected tilapia (**A2**), grass carp (**B2**), channel catfish (**C2**).

The diseased freshwater fish in this study exhibited obvious critical symptoms, including abnormal swimming behavior at the water surface, reduction in feed intake, pale or darker skin with or without ulcer formation, hemorrhage around their mouth, operculum, fin bases, fin erosion and swollen belly with hemorrhagic protrusion from the anal opening was also observed (Figure 1). In most of the inspected fish samples, gross lesions were mainly hemorrhagic and enlarged liver, gallbladder and spleen, empty or partially empty stomach and intestine, and enlarged and darkened posterior kidney. The impacted ponds/cages initially showed sporadic mortality and later mass mortality of infected fish.

### 2.2. Aeromonas Hydrophila Identification

A total of 458 bacterial isolates from diseased fish grew well on the Rimler–Shotts (RS) medium with small smooth and yellow colonies consisting of Gram-negative, rod-shaped, and motile bacilli were recovered and tentatively identified as *Aeromonas* species. The results of the phenotypic tests revealed that 338/458 isolates were identical to those of the reference *A. hydrophila* strain ATCC 7966 (Supplementary Table S1), accounted for 73.1% of the total isolates recovered from diseased fish.

**Figure 2:**
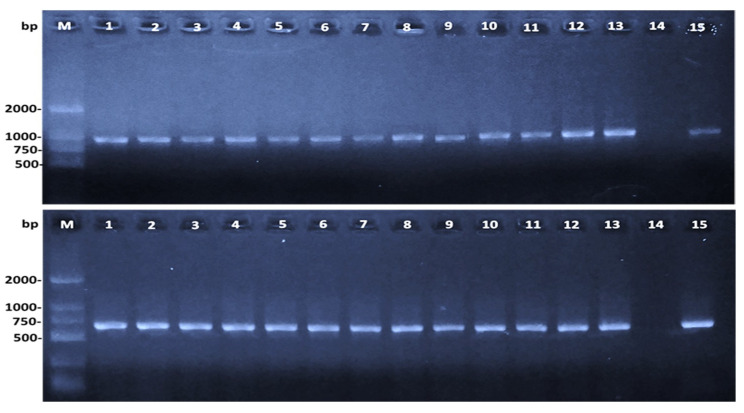
Amplified products of representative *A. hydrophila* isolates from tilapia (n = 5), carp (n = 4) and channel catfish (n = 4) of *Aeromonas* genus (953 bp)—upper ranges and *A. hydrophila* species (625 bp)—lower ranges: M: DNA ladder; lane 1–13 representative isolates from diseased fish; lane 14-negative control; lane 15-positive control (*A. hydrophila* ATCC 7966).

PCR assays successfully amplified both the 16S rRNA gene of *Aeromonas* genus (953 bp) and species-specific *A. hydrophila* gene (625 bp) of 255/338 isolates, including 102, 95 and 58 isolates from tilapia, carp, and channel catfish, respectively (Figure 2).

**Figure 3:**
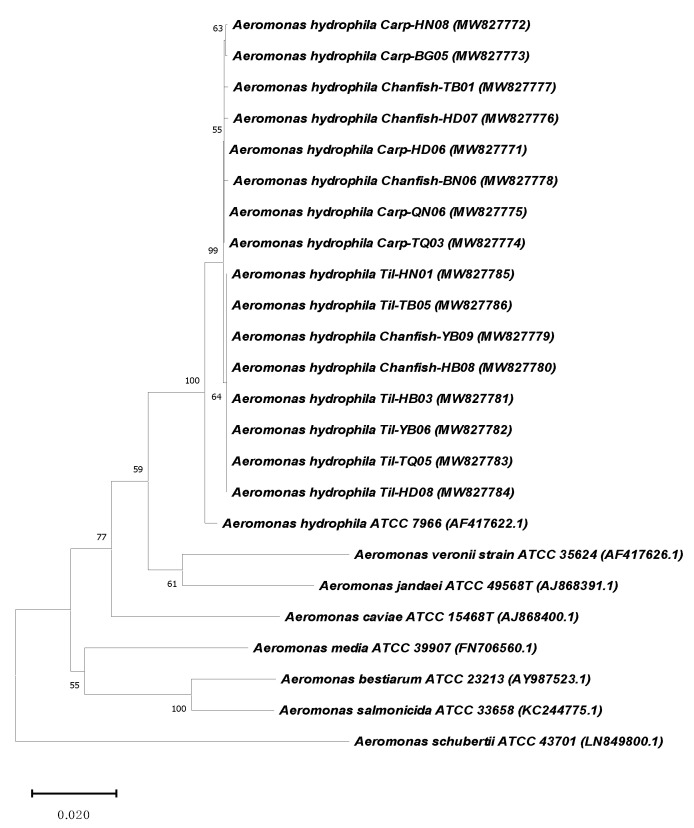
Phenotypic analysis based on *gyrB* sequences of the representative isolates recovered from the three fish hosts with those of other of *Aeromonas* species retrieved from Genbank using the neighbor-joining method. Bootstraps of 2000 replicates were performed.

**Figure 4:**
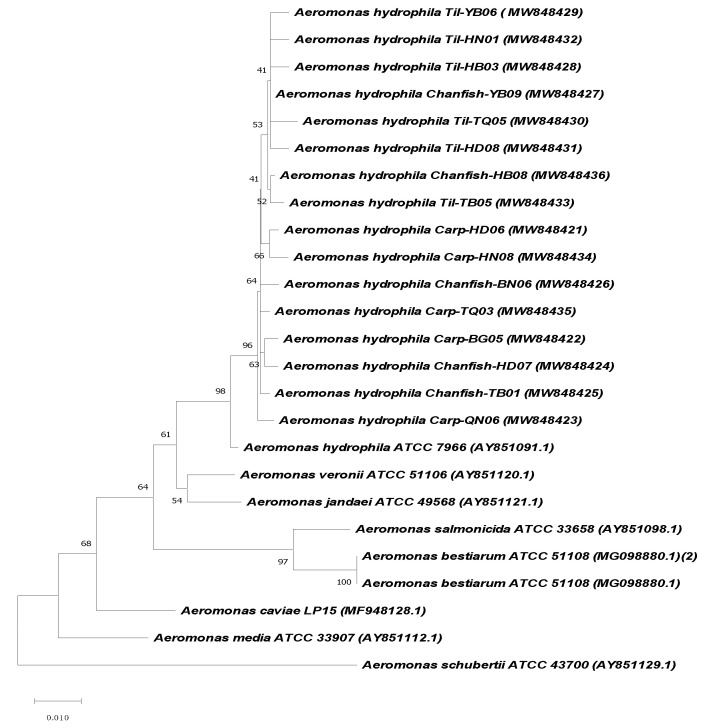
Phenotypic analysis based on *rpoB* sequences of the representative isolates recovered from the three fish hosts with those of other of *Aeromonas* species retrieved from Genbank using the neighbor-joining method. Bootstraps of 2000 replicates were performed.

The BLASTing analyses of *gyrB* and *rpoB* sequences revealed that 236/255 isolates were most precise identification as *A. hydrophila* due to their sequences shared the highest identities to those of other *A. hydrophila* in Genbank database and to those of the reference *A. hydrophila* strain ATCC 7966 (ranged 99.1–99.2%, and 98.4–99.1% for *gyrB* and *rpoB* sequences, respectively) (Table S2). Another 12 isolates with the sequence similarity below 97% (both or neither *gyrB* and/or *rpoB*) to the reference strain were subjected as misidentification. Due to a large number of isolates, the sequences of *gyrB* and *rpoB* genes from 16 representative isolates (including the highest and lowest sequence identities to those of *A. hydrophila* ATCC 7966) were submitted to Genbank under accession number MW827771-MW827786 (for *gyrB* genes), MW848421- MW848436 (for *rpoB* genes) and used for phylogenetic analysis. The derived neighbor-joining trees using *gyrB* and *rpoB* sequences in this study with other *Aeromonas* species in Genbank database revealed that the isolates of the present study are most closely related to *A. hydrophila* strain ATCC 7966 (Figure 3 and Figure 4). Based on the number of *A. hydrophila* isolates that were conclusively identified (236 isolates), the detection frequency of this pathogen was at 46.4%, on average, and no significant difference was shown in the frequencies of this bacteria detected on diseased tilapia, carp and channel catfish (*p* > 0.05) (Table 1).

### 2.3. Virulence Genes Characteristics

**Figure 5:**
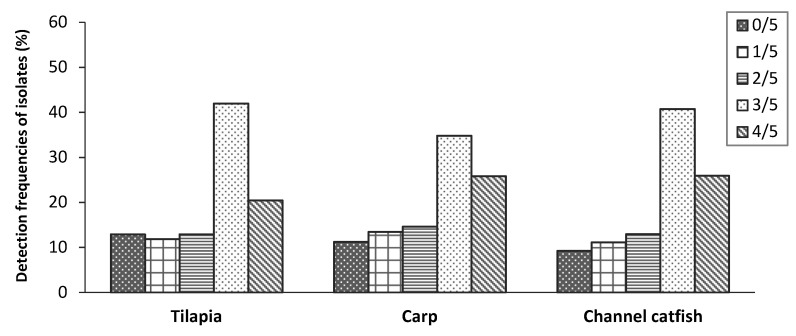
The detection frequencies of the *A. hydrophila* isolates from cultured tilapia, carp, and channel catfish carrying 0–4 virulence genes out of the 5 tested genes in the study.

**Figure 6:**
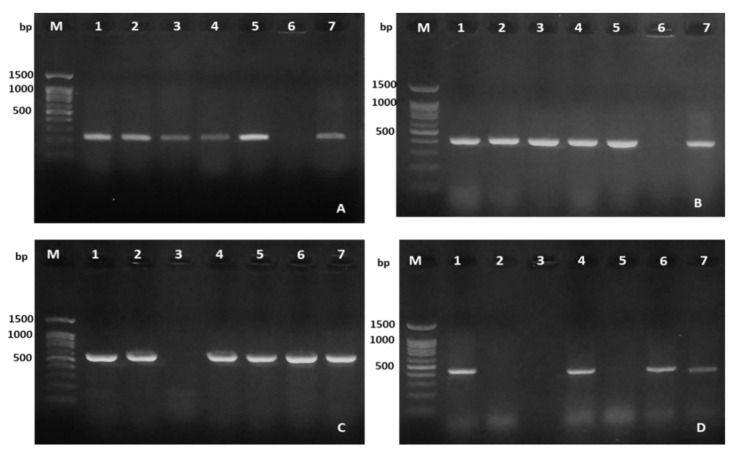
PCR amplification of virulence genes carried in representative *A. hydrophila* isolates. M: DNA ladder; lane 1–7 representative amplified products of *act* gene (232 bp) (**A**); *aerA* gene (431 bp) (**B**); *hlyA* gene (597 bp) (**C**); and *alt* gene (442 bp) (**D**).

The presence of the five putative virulence genes in *A. hydrophila* isolated from diseased fish samples is shown in Table 2 and Figure 5 and Figure 6. Of all isolates, no selected virulence gene was detected in 11.1% of the isolates while the other 88.9% isolates harbored at least one virulence gene. The Fisher’s exact test showed no significant difference in the detection frequency of each virulence gene on *A. hydrophila* isolates among the three fish species (*p* > 0.05). The highest frequencies of the isolates (39.2%) were found carrying 3/5 virulence genes and the proportion of isolates harboring 1/5, 2/5 and 4/5 virulence genes accounted for 12.1%, 13.5% and 24.1%, respectively. On average, aerolysin (*aerA*, 80.5%) and cytotoxic enterotoxin (*act*, 80.1%) were the most frequently detected virulence genes in *A. hydrophila* isolates from the three freshwater fish species. Hemolysin A (*hlyA*) was detected at a lower frequency of 59.7%, followed by the occurrence rates of heat-labile cytotonic enterotoxin (*alt*) gene 44.9%. In contrast, the heat-stable enterotoxin (*ast*) gene was not detected in any of the 236 isolates.

### 2.4. Antibiotic Susceptibility Patterns

Examination of antimicrobial susceptibility to 16 antimicrobials was performed on all 236 confirmed *A. hydrophila* isolates in this study. The results demonstrated that all isolates from tilapia, carp and channel catfish exhibited a serious and varying degree of resistance to all the tested antimicrobial agents.

Oxacillin (Ox), amoxicillin (Ax) and vancomycin (Va) showed the highest resistance frequencies (>70%) in *A. hydrophila* isolates from the three fish species. The resistance rate reached up to 100% for oxacillin and amoxicillin in the isolates from carp and channel catfish. The resistance ratio to neomycin (Ne) was approximately 80% of the isolates from tilapia, significantly higher than those of the isolates from carp (21.3%) and channel catfish (29.6%) (*p* < 0.05, Table 3 and Table 4).

All of the *A. hydrophila* isolates from channel catfish were susceptible to the six antimicrobial agents including cefotaxime (Ct), ceftriaxone (Cx), cefuroxime (Cu), ofloxacin (Of), norfloxacin (No), and doxycycline (Dx). A similar resistance outcome to cefotaxime was also detected in the isolates from carp and low resistance frequencies (<15%) were observed to the other five drugs. In contrast, the isolates from tilapia exhibited variable degrees of resistance to those antimicrobial agents at below 15% to cefuroxime, norfloxacin and doxycycline; and from 22.6 to 41.9% for cefotaxime, ceftriaxone and ofloxacin. All isolates expressed resistance frequencies below 15% to the combination of amoxicillin and clavulanic acid and slightly higher resistance frequencies (16.7–24.7%) were observed to florfenicol.

Relatively higher resistance ratios were found in *A. hydrophila* isolates from tilapia and carp to erythromycin (Er), nalidixic acid (Na) and oxytetracycline (OTC) (36.6–50.6%) compared to those of isolates from channel catfish (9.3–37.0%). The resistance rate of tilapia isolates to the combination of sulfamethoxazole and trimethoprim (SM/TM, 45.2%) was significantly higher than that of isolates from carp (22.2%) and channel catfish (24.7%), (*p* < 0.05).

**Figure 7:**
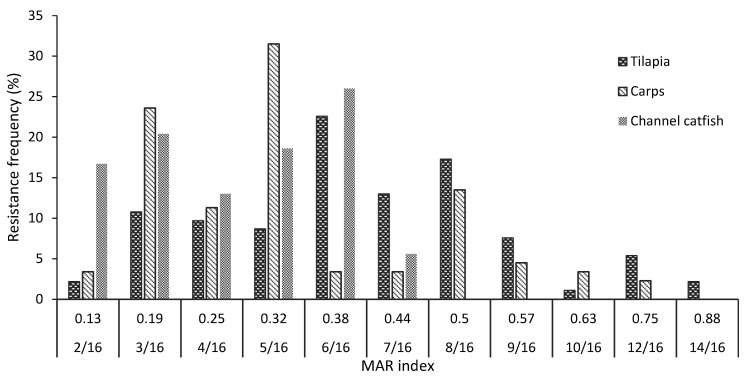
The distributions of multiple antibiotic resistances (MAR) of *A. hydrophila* isolates from cultured tilapia, carp, and channel catfish. None of the isolates was resistant to only 0 or 1 agent. The values 2/16 to 14/16 mean that the isolates showed resistance to 2 to 14 out of 16 antimicrobial agents with the corresponding MAR values.

The overall multiple antibiotic resistance (MAR) index ranged from 0.13 to 0.88, corresponding to 100% *A. hydrophila* isolates resistant to at least two antimicrobial agents. The highest MAR values of tilapia, carp, and channel catfish isolates were 0.88, 0.75, and 0.44, respectively (Figure 7). In total, 74.7% of the *A. hydrophila* isolates were observed with MAR ≥ 0.2 (87.6% of isolates from tilapia, followed by those of carp and channel catfish, at 73.3 and 63.2%, respectively). Almost 64% of the tilapia isolates were resistant to 6/16 to 8/16 antimicrobial agents, while 16.3% of the isolates showed resistance to more than 8 drugs (MAR > 0.5). On carp, more than 66% of the isolates resisted 3/16 to 5/16 antibiotics while more than 94% of the isolates from channel catfish resisted 2/16 to 6/16 of the antimicrobial agents.

## 3. Materials and Methods

### 3.1. Sample Locations and Clinical Examination of Diseased Fish

**Figure 8:**
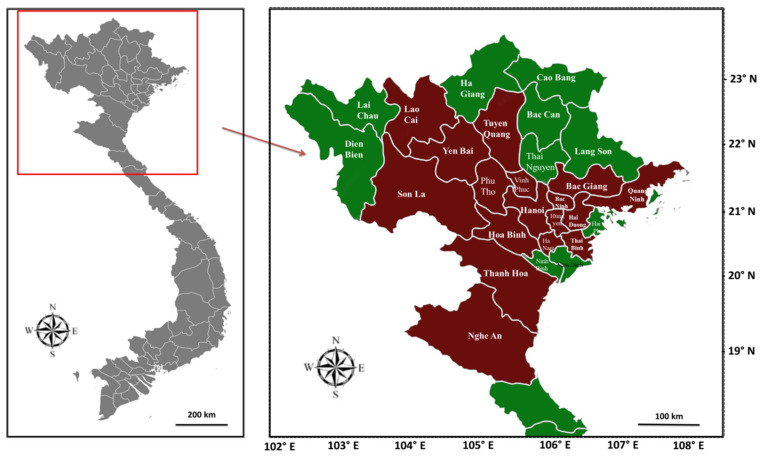
A map showing the provincial sites (indicated by red color) from which diseased fish were collected in the study.

In 2019–2020, a total of 506 diseased fish (moribund or recently died) of three cultured species including tilapia (198 samples), carp (mainly common carp and grass carp; 187 samples), and channel catfish (121 samples) were collected from 54 farms of the major culture areas located in sixteen provinces in Northern Vietnam (Figure 8). The fish samples were placed in sterile sealed plastic bags, transferred to the laboratory of the Department of Aquatic Environment and Fish Pathology—Faculty of Fisheries, Vietnam National University of Agriculture (VNUA) in a cold box (below 4 °C), and immediately analyzed. Clinical signs and gross features of all diseased fish were observed and recorded.

### 3.2. Bacterial Culture and Isolation

For detection of *A. hydrophila* in fish, the skin surface was first sterilized by swabbing with 70% ethyl alcohol before making dissection incisions. Samples of liver, kidney and spleen were streaked on Rimler–Shotts (RS) agar plates (Himedia, India), followed by incubation at 28 °C for 18–24 h. The presumptive colonies (yellow) were selected and re-isolated three times, followed by the determination of colony and bacterial morphology. Subsequently, isolates were stored in brain heart infusion (BHI) broth supplemented with 15% glycerol and kept at −80 °C for further examination.

### 3.3. Bacterial Identification

Conventional identification of *Aeromonas* species based on phenotypic methods is challenging due to the heterogeneous nature of the species [23]. Identification of *A. hydrophila* in the present study was, therefore, conducted using a combination of phenotypic tests and molecular methods. For phenotypic identification, the following tests were conducted including shape and color of colonies on Rimler–Shotts media, cell morphology, motility, catalase, cytochrome oxidase (OX), Voges–Proskauer (VP), hemolysis of sheep RBCs, sensitivity to 0/129 (150 µg), indole production (IND), urease production (URE), citrate utilization (CIT), glucose fermentation, H_2_S production, arginine dihydrolase (ADH), lysine decarboxylase (LDC), ornithine decarboxylase (ODC), melibiose fermentation (MEL), amygdalin fermentation (AMY), arabinose fermentation (ARA), inositol fermentation (INO), mannitol fermentation (MAN), tryptophane deaminase (TDA), gelatin hydrolysis (GEL). The isolates were selected if all test results were identical to those of the reference *A. hydrophila* strain ATCC 7966 (Supplementary Table S1).

For molecular identification, the genomic DNA of bacterial isolates was extracted using the InstaGene matrix (Bio-Rad laboratories, Hercules, CA, USA) according to the manufacturer’s protocol. All the suspected isolates which were screened by the phenotypic tests were subjected to PCR examination using primer sets to detect specific *Aeromonas* genus (953 bp) and *Aeromonas hydrophila* species (*AeroH*; 625 bp) as described in previous studies [24,25] (Table 5). PCR reaction mixtures and conditions were modified from previous study [25]. In brief, PCR reaction mixtures (total 20 µL) included 10 µL of Gotaq Green Master Mix (Promega, Madison, WI, USA), 1.5 µL of specific primer (each of forward and reverse), 3 µL DNA template and 4 µL of DNA-free distilled water. The mixtures were then placed in a thermal cycler for amplification under the following conditions: an initial denaturation of 4 min at 94 °C; 35 cycles consisting of denaturation at 95 °C for 30 s, annealing at 58 °C for 30 s, and extension step at 72 °C for 60 s; and a final extension for 7 min at 72 °C. The amplified products were then analyzed by electrophoresis on a 1% agarose gel containing Redsafe nucleic acid staining solution (Intron, Korea). The images were captured digitally using a Gel image system (Hercules, CA, USA).

Due to the complexity of *Aeromonas* species identification, the specific genes sequence analyses were conducted after PCR examination [26]. The DNAs of the PCR-positive isolates were used for sequencing the housekeeping genes *gyrB* (~1100 pb) and *rpoB* (560 bp) with the corresponding primer sets presented in Table 5. The sequences of *gyrB* and *rpoB* genes of the suspected *A. hydrophila* isolates were evaluated the identity to other *Aeromonas* species and *A. hydrophila* ATCC 7966 from the Genbank database. The representative sequences of *gyrB* and *rpoB* genes and those of other *Aeromonas* species were aligned using ClustalW [27], and phylogenetic trees were then constructed using the neighbor-joining method [28] in Mega X software [29].

### 3.4. Virulence Genes Detection

The presence of the genes encoding enterotoxins (*act, alt, ast*) and hemolysins (*hlyA* and *aerA*) in the genome of *A. hydrophila* isolates was detected by PCR using specific primers described in previous studies [32,33,34] (Table S3). The protocols of DNA extraction and PCR reaction mixture were similar to those used in bacterial identification on the corresponding primers. The thermal conditions consisted of an initial denaturation of 94 °C for 3 min; followed by 35 cycles of amplification, each consisting of 94 °C denaturation for 30 s, annealing for 50 s at 1 °C below the lowest Tm of a given primer pair reported by the previous authors (Table S3); and 72 °C extension for 10 min. The PCR products were then subjected to electrophoresis, visualized, and photographed in a similar manner to the protocol used for bacterial identification.

### 3.5. Antimicrobial Susceptibility Testing

The susceptibility of *A. hydrophila* isolates to antibiotics was examined using the disc diffusion technique according to the guidelines of the Clinical Laboratory Standards Institute (CLSI) [35]. Sixteen antibiotics of 11 antibiotic classes/subclasses were tested including two penicillins: oxacillin (Ox, 1 µg) and amoxicillin (Ax, 10 µg); one β-Lactam/β-Lactamase inhibitor combination (BL/BLIs): amoxicillin-clavulanic acid (Ac, 20/10 µg); three cephalosporins: cefotaxime (Ct, 30 µg), cefuroxime (Cu, 30 µg), and ceftriaxone (Cx, 30 µg); one macrolide: erythromycin (Er, 15 µg); one quinolone: nalidixic acid (Na, 30 µg); one sulfonamide: sulfamethoxazole/trimethoprim (SM/TM, 23.75/1.25 µg); one aminoglycosides: neomycin (Ne, 30 µg); one glycopeptide: vancomycin (Va, 30 µg); two fluoroquinolones: ofloxacin (Of, 5 µg) and norfloxacin (No, 10 µg); two tetracyclines: doxycycline (Dx, 30 µg) and oxytetracycline (OTC, 30 µg); and one amphenicol: florfenicol (Fl, 30 µg). The selected antibiotics are the commonly used drugs or highly detected frequencies in aquatic environments surrounding the aquaculture areas in Vietnam [36,37,38,39].

*Aeromonas hydrophila* isolates were grown in Mueller–Hinton (MH) broth and were adjusted to reach a McFarland turbidity of 0.5. The suspension was then spread onto Mueller–Hinton agar by a sterilized cotton swab. Antibiotic discs were placed onto the inoculated plates and incubated at 28 °C. The zones of inhibition were recorded and classified as susceptible, intermediate, and resistant according to zone diameter interpretation standards described in the CLSI M100-S25 [35]. Since as the CLSI breakpoint for *A. hydrophila* includes only a few antimicrobial agents, CLSI breakpoints for *Enterobacteria* were instead used. The multiple antibiotic resistance (MAR) index of the isolates was calculated as described by Krumperman [40], in which MAR = a/b, where ‘a’ represents the number of antibiotics to which the isolate was resistant and ‘b’ represents the total number of antibiotics to which the isolates were exposed for susceptibility testing.

### 3.6. Data Analysis

The prevalence of infection, detection frequencies of virulence genes and antibiotic resistance ratios of *A. hydrophila* isolated from the three fish species (tilapia, carp, and channel catfish) were compared with Fisher’s exact test using SPSS software (version 20). For all the analyses, statistical significance was determined if a two-tailed *p*-value was no more than 0.05.

## 4. Discussion

Freshwater fish farming has accounted for almost 50% of the world’s aquaculture production [1]. Of this, tilapia, carp and catfish represent the primary cultured species globally and in Northern Vietnam [41]. The farming systems of these species have recently been shifted toward intensive modes characterized by a high organic matter load and high stocking densities, which are the favorable conditions for bacterial disease outbreaks. In the present study, 46.4% of diseased fish with the typical hemorrhagic septicemia symptoms were infected with *A. hydrophila*, which reveals the predominance of this pathogen in freshwater fish farming in Northern Vietnam and constitutes a huge economic loss due to the high accumulative mortality of fish in impacted ponds/cages. The current study showed no significant difference in detection frequencies of *A. hydrophila* among diseased tilapia, carp, and channel catfish. The development of cage culture with multiple species in open systems (rivers, lakes) and/or inadequate water management in earth ponds probably caused similar infection frequencies between fish, creating favorable conditions to spread this pathogen among fish farms.

*Aeromonas hydrophila* has emerged as an important foodborne pathogen worldwide [42,43]. The consumption of contaminated water and aquatic food products are considered the main sources of human *Aeromonas* infection [44]. *A. hydrophila* can cause gastrointestinal as well as extra-intestinal infections in humans [45,46]. There is evidence demonstrated the relationship between the presence of virulence-associated factors and the virulence increase in fish and other hosts [47]. In the present study, 88.9% of the *A. hydrophila* isolates from diseased fish carried the tested virulence genes and 39.2% of isolates carried 3/5 virulence genes. The result indicates that most of the *A. hydrophila* isolates from diseased tilapia, carp and channel catfish had a high potential pathogenicity on fish and potentially on fish consumers. Their infection is likely responsible for hemorrhagic septicemia symptoms in the fish hosts. However, although all the fish infected with *A. hydrophia* in this study exhibited similar clinical symptoms and gross lesions, 11.1% of the isolates had none of the tested virulence genes. The results suggest for these isolates the possible involvement of other virulence genes and pathogenic factors not investigated in this study, such as genes encoding proteases and lipase, in inducing hemorrhagic septicemia symptoms in diseased fish [34,48], or the concurrence of infection with other pathogens [49].

The heterogeneity in the frequencies of the virulence gene among *A. hydrophila* isolates in the present and previous studies demonstrates the variability of virulence gene profiles of the isolates. The present study showed that aerolysin *aerA* and cytotoxic enterotoxin *act* genes (harbored by 80.5%, and 80.1% of isolates, respectively) were most prevalent. Gene *aerA* codes a pore-forming toxin that binds to receptors on the target cell membrane while *act* has enterotoxic, hemolytic, and cytotoxic activities and involved in tissue damage and fluid secretion in intestinal epithelial cells of infected fish [17,50]. The high prevalence of *aerA* and *act* genes was also reported by previous studies [51,52]. Hemolysin *hlyA* is another important virulence factor, not only in lysing red blood cells but in cytotoxic activity against a broad range of species and cell types [53,54]. In the current work, the *hlyA* gene was detected in 57.5 to 63.0% of isolates which is higher than reported by El-Bahar et al. at 9.09% of *A. hydrophila* isolates from diseased tilapia in Egypt [51]. Meanwhile, Hayati et al. recorded a very high distribution of *hlyA* gene (95%) in this pathogen from farmed and wild tilapia, climbing perch and catfish sampled in Malaysia [55]. Heat labile enterotoxin (*alt*) contributes to promoting fluid accumulation in the small intestine of animals [56]. Our study showed 42.5% of the isolates carrying the *alt* gene which was lower than those reported by Rather et al. [57] on *A. hydrophila* isolated from various fish species. Heat stable cytotonic enterotoxin *ast* causes CHO cells to elongate and evokes intestinal fluid accumulation [56]. However, in the present study, the *ast* gene was absent in all *A. hydrophila* isolates, in contrast to 70% of *A. hydrophila* isolates from tilapia and channel catfish in Egypt carrying the *ast* gene [58]. The variabilities in detection frequencies of virulence genes among *A. hydrophila* isolates might be due to the geographical distribution of strains and the possibility of horizontal gene transfer [21,59].

Assessments of antimicrobial susceptibility are important to monitor the severity of antimicrobial resistance and to select proper drugs for disease treatments in fish farming, minimizing the risk to human health. In intensive farming systems, there is a widespread and often unregulated use of antimicrobial agents to control infectious diseases [4], resulting in the emergence of reservoirs of antimicrobial-resistant bacteria in fish and other aquatic animals, as well as in the aquatic environment [60,61]. From these reservoirs, resistance genes may be disseminated by horizontal gene transfer and reach human pathogens, or drug-resistant pathogens from the aquatic environment may reach humans directly [62]. The severity of antimicrobial resistance in *A. hydrophila* has been reported in various cultured species [63]. In the present study, 100% *A. hydrophila* isolates from tilapia, carp and channel catfish displayed variable degrees of antimicrobial resistance to the 16 agents of 11 antibiotic subclasses and most of the isolates were resistant to multiple antimicrobial agents. This is the first study carried out on a wide range of important cultured freshwater fish in the Northern Vietnam where the intensive farming systems have been expanded rapidly in recent years. The result reveals that antibiotic resistance of *A. hydrophila* in freshwater fish farming in Vietnam is close to the situation occurring in many other countries and adds to the global concerns.

Despite the divergences in the susceptibility patterns of the *A. hydrophila* isolates to the tested antibiotics, the tilapia isolates exhibited an overall higher degree of resistance to those drugs. The highest resistance rates of *A. hydrophila* isolates from freshwater fish species were observed for penicillins (oxacillin and amoxicillin) group (>90%) and vancomycin (>73%). The present result is consistent with previous reports and has been explained due to the intrinsic resistance of *Aeromonas* spp. to beta-lactams and glycopeptide antibiotics [64,65]. Although most of the isolates were resistant to penicillins, very low resistance frequencies of *A. hydrophila* isolates were shown for the combination of amoxicillin and clavulanic acid (AC) because of the capacity of clavulanic acid for beta-lactamase inhibition, preventing bacteria from destroying amoxicillin [46]. Significantly lower resistance frequencies (*p* < 0.001) of *A. hydrophila* isolates from carp and channel catfish (21.3–29.6%) to neomycin were detected in comparison to that of isolates from tilapia (80%). Regarding the Cephem subclass, most isolates from carp and channel catfish were susceptible to the second and the third cephalosporins (Ct, Cu and Cx), but a reduced susceptibility has been shown on tilapia isolates to these agents. Although *Aeromonas* spp. are generally susceptible to later-generation cephalosporins [21,66], the increased resistance to the second- and third-generation cephalosporins has been observed in clinical and seafood isolates [15]. High susceptibility of the isolates from carp and channel catfish and lower susceptibility of the tilapia isolates were also observed for fluoroquinolones (Of and No) and sulfonamides (SM/TM). The dissimilarity in resistance patterns of *A. hydrophila* isolates among fish species might imply historical differences in the use of antimicrobial agents in different fish culture system in terms of frequencies, quantity, utilization methods and drug species [67]. Similar phenomena of the high variability in antibiotic resistance frequencies on *Aeromonas* spp. from a diversity of fish species in different geographical regions were also previously reported [65,68].

The multiple antibiotic resistance (MAR) index has been used to indicate the degrees of antibiotic utilization. A MAR index value higher than 0.2 reflects the bacterial isolates from high-risk sources of antibiotic contamination where antibiotics are often used [40]. The present study showed an average of 74.7% *A. hydrophila* isolates from the three fish species having a MAR value higher than 0.2, with the highest frequency (87.6%) of the isolates from tilapia, followed by that of the isolates from carp (73.3%) and channel catfish (63.3%). The result demonstrates the overall high prevalence of multiple antibiotic resistances in *A. hydrophila* isolates from freshwater fish in Vietnam. The higher frequencies of antibiotic resistance in tilapia isolates in comparison to those from carp and channel catfish might imply the more frequent utilization of antimicrobial agents in tilapia farming.

## 5. Conclusions

The infection prevalence of *Aeromonas hydrophila* has been determined at 46.4% associated with freshwater fish disease in Northern Vietnam. Most *A. hydrophila* isolates harbored at least one of the five virulence genes (*aerA, hlyA, alt, act,* and *ast*) that posed a high potential pathogenicity to fish mortality. The majority of *A. hydrophila* isolates in the present study showed multiple antibiotic resistances when exposed to 16 antibiotics. These results might be a warning to farmers and aquaculture managers regarding to mitigating the spread of *A. hydrophila* in culture systems, enhance the awareness of the appropriate uses of antibiotics in fish farms. In addition, other environmentally friendly treatments alternatives such as herbal therapy, phages, and vaccination should be encouraged to apply to reduce the antibiotic resistance, improve food quality, and minimize negative impacts to human and environment.

## Figures and Tables

**Table 1:** Prevalence of *A. hydrophila* isolated from diseased tilapia, carp and channel catfish.

Fish Species	Total no. of Samples	Number of Isolates				
		Recovered on Rimler–Shotts Medium	Confirmed by Phenotypic Tests	Confirmed by PCR	Confirmed bySequencing	Detection Frequencies (%)
Tilapia	198	187	136	102	93	47.0
Carp	187	156	118	95	89	47.6
Channel catfish	121	115	84	58	54	44.6
**Total/mean**	**506**	**458**	**338**	**255**	**236**	**46.4**

**Table 2:** Detection frequencies of the five virulence genes in *A. hydrophila* isolated from tilapia, carp and channel catfish.

Virulence Genes	Detection Frequencies % (N)			
	Tilapia (n = 93)	Carp (n = 89)	Channel Catfish (n = 54)	Mean (n = 236)
*hlyA*	58.1 (54)	63.0 (56)	57.5 (31)	59.7 (141)
*aerA*	83.9 (78)	79.8 (71)	76.0 (41)	80.5 (190)
*act*	83.9 (78)	76.5 (68)	79.7 (43)	80.1 (189)
*alt*	42.0 (39)	47.2 (42)	46.3 (25)	44.9 (106)
*ast*	0 (0)	0 (0)	0 (0)	0 (0)

**Table 3:** Antimicrobial susceptibility profiles of *A. hydrophila* isolates from tilapia, carp and channel catfish.

Antimicrobial Agents	Antimicrobial Susceptibility of *A. hydrophila* (N = 236)								
	Tilapia (n = 93)			Carp (n = 89)			Channel Catfish (n = 54)		
	S (%)	I (%)	R (%)	S (%)	I (%)	R (%)	S (%)	I (%)	R (%)
**Penicillins (PNs)**									
Oxacillin (Ox)	0 (0)	4.4 (4)	95.7 (89)	0 (0)	0 (0)	100 (89)	0 (0)	9.3 (5)	90.8 (49)
Amoxicillin (Ax)	15.1 (14)	7.6 (7)	77.5 (72)	3.4 (3)	0(0)	96.7 (86)	0 (0)	0 (0)	100 (54)
**Β-Lactam/β-Lactamase inhibitor combination (BL/BLI)**									
Amoxicillin-Clavulanic acid (AC)	76.4 (71)	15.1 (14)	8.7 (8)	73.1 (65)	18 (16)	9.0 (8)	68.6 (37)	16.7 (9)	14.9 (8)
**Cephems (CPs)**									
Ceftriaxone (Ct)	69.9 (65)	4.4 (4)	25.9 (24)	95.6 (85)	4.5 (4)	0 (0)	90.8 (49)	9.3 (5)	0(0)
Cefuroxime (Cu)	81.8 (76)	5.4 (5)	13.0 (12)	96.7 (86)	0 (0)	3.4 (3)	100 (54)	0(0)	0(0)
Cefotaxime (Cx)	77.5 (72)	0 (0)	22.6 (21)	96.7 (86)	0 (0)	3.4 (3)	100 (54)	0(0)	0(0)
**Macrolides (MCs)**									
Erythromycin (Er)	24.8 (23)	32.3 (30)	43.1 (40)	38.3 (34)	14.7 (13)	47.2 (42)	57.5 (31)	33.4 (18)	9.3 (5)
**Quinolones (QLs)**									
Nalidixic (Na)	54.9 (51)	0 (0)	45.2 (42)	46.1 (41)	3.4 (3)	50.6 (45)	53.8 (29)	9.3 (5)	37.1 (20)
**Sulfonamides (SULs) (Folate pathway inhibitors)**									
Sulfamethoxzole- Trimethoprim (SM/TM)	36.6 (34)	18.3 (17)	45.2 (42)	75.3 (67)	0 (0)	24.8 (22)	68.6 (37)	9.3 (5)	22.3 (12)
**Aminoglycosides (AMGs)**									
Neomycin (Ne)	4.4 (4)	16.2 (15)	79.6 (74)	42.7 (38)	36.0 (32)	21.4 (19)	38.9 (21)	31.5 (17)	29.7 (16)
**Glycopeptide (GLs)**									
Vancomycin (Va)	3.3 (3)	15.1 (14)	81.8 (76)	6.8 (6)	20.3 (18)	73.1 (65)	0 (0)	16.7 (9)	83.4 (45)
**Flouroquinolones (FQNs)**									
Ofloxacin (Of)	49.5 (46)	8.7 (8)	42 (39)	79.8 (71)	3.4 (3)	16.9 (15)	61.2 (33)	38.9 (21)	0 (0)
Norfloxacin (No)	63.5 (59)	24.8 (23)	11.9 (11)	83.2 (74)	3.4 (3)	13.5 (12)	77.8 (42)	22.3 (12)	0 (0)
**Tetracyclines (TCs)**									
Doxycycline (Dx)	88.2 (82)	9.7 (9)	2.2 (2)	75.3 (67)	18 (16)	6.8 (6)	100 (54)	0 (0)	0(0)
Oxytetracycline (OTC)	59.2 (55)	4.4 (4)	36.6 (34)	55.1 (49)	0 (0)	45 (40)	70.4 (38)	0 (0)	29.7 (16)
**Amphenicols (AMPs)**									
Florfenicol (Fl)	72.1 (67)	3.3 (3)	24.8 (23)	76.5 (68)	0 (0)	23.6 (21)	83.4 (45)	0 (0)	16.7 (9)
**Overall**									
Average (%)	48.5	10.6	40.9	59.0	7.6	33.4	60.6	12.3	27.1

R = resistant; I = intermediate; S = susceptibility; n = number of isolates from each source; N: total number of isolates.

**Table 4:** Antimicrobial-resistant phenotypes of the isolates confirmed from tilapia, carp and channel catfish.

No of Drugs	Resistance Phenotypes	The Ratio of Isolates—% (N)		
		Tilapia n = 93	Carp n = 89	Channel Catfish n = 54
2	Ox + Ax	2.2 (2)	3.4 (3)	16.7 (9)
3	Ox + Ne + Va	7.6 (7)	0 (0)	0 (0)
	Ox + Ax + Ne	3.3 (3)	0 (0)	0 (0)
	Ox + Ax + Na	0 (0)	2.3 (2)	0 (0)
	Ox + Ax + Va	0 (0)	21.4 (19)	20.4 (11)
4	Ox + Ax + Na + Va	4.4 (4)	3.4 (3)	13 (7)
	Ox + Ax + Of + Ne	3.3 (3)	3.4 (3)	0 (0)
	Ox + Ax + Ne + Va	2.2 (2)	0 (0)	0 (0)
	Ox + Ax + Va + Fl	0 (0)	4.5 (4)	0 (0)
5	Ox + Ax + Na + Ne + Va	3.3 (3)	0 (0)	11.2 (6)
	Ox + Ax + Er + Ne + Va	5.4 (5)	5.7 (5)	0 (0)
	Ox + Ax + SM/TM + Fl + OTC	0 (0)	3.4 (3)	0 (0)
	Ox + Ax + Na + Va + OTC	0 (0)	3.4 (3)	7.5 (4)
	Ox + Ax + SM/TM + Va + OTC	0 (0)	1.2 (1)	0 (0)
	Ox + Ax + Er + Na + OCT	0 (0)	18 (16)	0 (0)
6	Ox + Ct + Cx + Va + Fl + OTC	5.4 (5)	0 (0)	0 (0)
	Ox + Ax + Er + SM/TM + Ne + Va	3.3 (3)	0 (0)	0 (0)
	Ox + Ax + SM/TM + Ne + Va + OTC	3.3 (3)	3.4 (3)	0 (0)
	Ox + Ax + Er + Na + Ne + OTC	2.2 (2)	0 (0)	0 (0)
	Ox + Ax + Cu + SM/TM + Ne + Va	3.3 (3)	0 (0)	0 (0)
	Ox + Ax + Ac + Er + Ne + Va	4.4 (4)	0 (0)	9.3 (5)
	Ox + Ax + Er + Of + Ne + Va	1.1 (1)	0 (0)	0 (0)
	Ox + Ax + SM/TM + Va + Fl + OTC	0 (0)	0 (0)	7.5 (4)
	Ax + SM/TM + Ne + Va + Fl + OTC	0 (0)	0 (0)	9.3 (5)
7	Ox + Ax + Ct + Er + Ne + Va + OTC	3.3 (3)	0 (0)	0 (0)
	Ox + Ax + SM/TM + Ne + Va + Fl + OTC	2.2 (2)	0 (0)	0 (0)
	Ox + Ax + Na + SM/TM + Ne + Va + Fl	3.3 (3)	0 (0)	0 (0)
	Ox + Ax + Na + Of + Ne + Va + OTC	4.4 (4)	0 (0)	0 (0)
	Ox + Ax + Ac + Na + Of + No + Va	0 (0)	3.4 (3)	0 (0)
	Ox + Ax + Ac + Na + SM/TM + Va + OTC	0 (0)	0 (0)	5.6 (3)
8	Ox + Ct + Cx + Cu + Of + Ne + Va + OTC	2.2 (2)	0 (0)	0 (0)
	Ox + Ct + Cx + Na + Of + SM/TM + Ne + OTC	3.3 (3)	0 (0)	0 (0)
	Ox + Ax + Er + Na + Of + SM/TM + Ne + Va	5.4 (5)	0 (0)	0 (0)
	Ox + Ax + Er + Na + Of + No + SM/TM + Va	5.4 (5)	4.5 (4)	0 (0)
	Ox + Ax + Ac + Er + Na + Of + Ne + Va	1.1 (1)	3.4 (3)	0 (0)
	Ox + Ax + Er + No + SM/TM + Va + Fl + OTC	0 (0)	3.4 (3)	0 (0)
	Ox + Ax + Er + Na + SM/TM + Va + Fl + OTC	0 (0)	2.3 (2)	0 (0)
9	Ct + Cx + Na + Of + No + SM/TM + Ne + Fl + OTC	4.4 (4)	0 (0)	0 (0)
	Ox + Ax + Er + Na + Of + SM/TM + Va + Fl + OTC	3.3 (3)	0 (0)	0 (0)
	Ox + Ax + Er + Na + SM/TM + Va + Dx + Fl + OTC	0 (0)	4.5 (4)	0 (0)
10	Ox + Ax + Er + Na + Of + SM/TM + Ne + Va + Fl + OTC	1.1 (1)	0 (0)	0 (0)
	Ox + Ax + Cu + Cx + Er + Na + Ne + Va + Fl + OTC	0 (0)	3.4 (3)	0 (0)
12	Ox + Ax + Ct + Cu + Cx + Er + Na + Of + SM/TM + Ne + Va + Fl	2.2 (2)	0 (0)	0 (0)
	Ox + Ax + Ac + Ct + Cu + Cx + Er + Of + SM/TM + Ne + Va + Fl	3.3 (3)	0 (0)	0 (0)
	Ox + Ax + Ac + Er + Na + Of + No + SM/TM + Ne + Va + Fl + OTC	0 (0)	2.3 (2)	0 (0)
14	Ox + Ax + Ct + Cu + Cx + Er + Na + Of + No + SM/TM + Ne + Va + Dx + OTC	2.2 (2)	0 (0)	0 (0)

**Table 5:** Primers used for *A. hydrophila* identification in this study.

Gene	Primers	DNA Sequence (5′→3′)	Product Size (bp)	References
*16S rRNA*	Aero16S-F	CTACTTTTGCCGGCGAGCGG TGATTCCCGAAGGCACTCCC	953	[24]
	Aero16S-R			
*AeroH*	AeroH-F	GAAAGGTTGATGCCTAATACGTA	625	[25]
	AeroH-R	CGTGCTGGCAACAAAGGACAG		
*gyrB*	gyrB 3F	TCCGGCGGTCTGCACGGCGT	1110	[30]
	gyrB 14R	TTGTCCGGGTTGTACTCGTC		
*rpoB*	PasrpoB-L	GCAGTGAAAGARTTCTTTGGTTC	560	[31]
	RpoB-R	GTTGCATGTTNGNACCCAT		

## Data Availability

No new data available.

## Supplementary Materials

The following are available online at https://www.mdpi.com/article/10.3390/antibiotics10050532/s1, Table S1 Biochemical characterization of *A. hydrophila* in the present study; Table S2: The identities of *gyrB* and *rpoB* sequences from *A. hydrophila* isolates in present study and those of *A. hydrophila* ATCC ATCC 7966; Table S3: Primers used for the detection of virulence genes in *A. hydrophila.*

## References

[1] Davis D., Nguyen T., Li M., Gatlin D.M., O’Keefe T., Burnell G., Allan G. (2009). Advances in aquaculture nutrition: Catfish, tilapia and carp nutrition. New Technologies in Aquaculture.

[2] Eissa A., Moustafa M., El-Husseiny I., Saeid S., Saleh O., Borhan T. (2009). Identification of some skeletal deformities in freshwater teleosts raised in Egyptian aquaculture. Chemosphere.

[3] Mzula A., Wambura P.N., Mdegela R.H., Shirima G.M. (2020). Present status of aquaculture and the challenge of bacterial diseases in freshwater farmed fish in Tanzania; A call for sustainable strategies. Aquac. Fish..

[4] Heuer O.E., Kruse H., Grave K., Collignon P., Karunasagar I., Angulo F.J. (2009). Human health consequences of use of antimicrobial agents in aquaculture. Clin. Infect. Dis..

[5] Tavares-Dias M., Martins M.L. (2017). An overall estimation of losses caused by diseases in the Brazilian fish farms. J. Parasit. Dis..

[6] Dias M.K., Sampaio L.S., Proietti-Junior A.A., Yoshioka E.T., Rodrigues D.P., Rodriguez A.F., Ribeiro R.A., Faria F.S., Ozório R.O., Tavares-Dias M. (2016). Lethal dose and clinical signs of *Aeromonas hydrophila* in *Arapaima gigas* (Arapaimidae), the giant fish from Amazon. Vet. Microbiol..

[7] Papadopoulou C., Economou E., Zakas G., Salamoura C., Dontorou C., Apostolou J. (2007). Microbiological and pathogenic contaminants of seafood in Greece. J. Food Qual..

[8] Janda J.M., Abbott S.L. (2010). The genus *Aeromonas*: Taxonomy, pathogenicity, and infection. Clin. Microbiol. Rev..

[9] Plumb J.A., Hanson L.A. (2010). Health Maintenance and Principal Microbial Diseases of Cultured Fishes.

[10] Abdel-Latif H.M., Khafaga A.F. (2020). Natural co-infection of cultured Nile tilapia *Oreochromis niloticus* with *Aeromonas hydrophila* and *Gyrodactylus cichlidarum* experiencing high mortality during summer. Aquac. Res..

[11] Abd-El-Malek A.M. (2017). Incidence and virulence characteristics of *Aeromonas* spp. in fish. Vet. World.

[12] Kumar R., Pande V., Singh L., Sharma L., Saxena N., Thakuria D., Singh A.K., Sahoo P.K. (2016). Pathological findings of experimental *Aeromonas hydrophila* infection in golden mahseer (Tor putitora). Fish Aquac. J..

[13] Zhang D., Moreira G.S., Shoemaker C., Newton J.C., Xu D.-H. (2016). Detection and quantification of virulent *Aeromonas hydrophila* in channel catfish tissues following waterborne challenge. FEMS Microbiol. Lett..

[14] Palu A.P., Gomes L.M., Miguel M.A.L., Balassiano I.T., Queiroz M.L.P., Freitas-Almeida A.C., de Oliveira S.S. (2006). Antimicrobial resistance in food and clinical *Aeromonas* isolates. Food Microbiol..

[15] Lee H.J., Hoel S., Lunestad B.T., Lerfall J., Jakobsen A.N. (2020). *Aeromonas* spp. isolated from ready-to-eat seafood on the Norwegian market: Prevalence, putative virulence factors and antimicrobial resistance. J. Appl. Microbiol..

[16] Albert M.J., Ansaruzzaman M., Talukder K.A., Chopra A.K., Kuhn I., Rahman M., Mollby R. (2000). Prevalence of enterotoxin genes in *Aeromonas* spp. isolated from children with diarrhea, healthy controls, and the environment. J. Clin. Microbiol..

[17] Sha J., Kozlova E.V., Chopra A.K. (2002). Role of various enterotoxins in *Aeromonas hydrophila*-induced gastroenteritis: Generation of enterotoxin gene-deficient mutants and evaluation of their enterotoxic activity. Infect. Immun..

[18] Rico A., Phu T.M., Satapornvanit K., Min J., Shahabuddin A., Henriksson P.J., Murray F.J., Little D.C., Dalsgaard A., Van den Brink P.J. (2013). Use of veterinary medicines, feed additives and probiotics in four major internationally traded aquaculture species farmed in Asia. Aquaculture.

[19] Le T.X., Munekage Y. (2004). Residues of selected antibiotics in water and mud from shrimp ponds in mangrove areas in Viet Nam. Mar. Pollut. Bull..

[20] Rhodes G., Huys G., Swings J., Mcgann P., Hiney M., Smith P., Pickup R.W. (2000). Distribution of oxytetracycline resistance plasmids between *aeromonads* in hospital and aquaculture environments: Implication of Tn1721 in dissemination of the tetracycline resistance determinant Tet A. Appl. Environ. Microbiol..

[21] Yano Y., Hamano K., Tsutsui I., Aue-umneoy D., Ban M., Satomi M. (2015). Occurrence, molecular characterization, and antimicrobial susceptibility of *Aeromonas* spp. in marine species of shrimps cultured at inland low salinity ponds. Food Microbiol..

[22] Rabobank, World Seafood Trade Map 2019. https://research.rabobank.com/far/en/sectors/animal-protein/world-seafood-trade-map.html.

[23] Hoai T.D., Trang T.T., Van Tuyen N., Giang N.T.H., Van Van K. (2019). *Aeromonas veronii* caused disease and mortality in channel catfish in Vietnam. Aquaculture.

[24] Lee C., Cho J.C., Lee S.H., Lee D.G., Kim S.J. (2002). Distribution of *Aeromonas* spp. as identified by 16S rDNA restriction fragment length polymorphism analysis in a trout farm. Appl. Microbiol..

[25] Nielsen M.E., Hoi L., Schmidt A.S., Qian D., Shimada T., Shen J.Y., Larsen J.L. (2001). Is *Aeromonas hydrophila* the dominant motile *Aeromonas* species that causes disease outbreaks in aquaculture production in the Zhejiang Province of China?. Dis. Aquat. Organ..

[26] Samayanpaulraj V., Sivaramapillai M., Palani S.N., Govindaraj K., Velu V., Ramesh U. (2020). Identification and characterization of virulent *Aeromonas hydrophila* Ah17 from infected *Channa striata* in river Cauvery and in vitro evaluation of shrimp chitosan. Food Sci. Nutr..

[27] Thompson J.D., Higgins D.G., Gibson T.J. (1994). CLUSTAL W: Improving the sensitivity of progressive multiple sequence alignment through sequence weighting, position-specific gap penalties and weight matrix choice. Nucleic Acids Res..

[28] Saitou N., Nei M. (1987). The neighbor-joining method: A new method for reconstructing phylogenetic trees. Mol. Biol. Evol..

[29] Kumar S., Stecher G., Li M., Knyaz C., Tamura K. (2018). MEGA X: Molecular evolutionary genetics analysis across computing platforms. Mol. Biol. Evol..

[30] Yanez M., Catalán V., Apráiz D., Figueras M., Martinez-Murcia A. (2003). Phylogenetic analysis of members of the genus *Aeromonas* based on *gyrB* gene sequences. Int. J. Syst. Evol. Microbiol..

[31] Korczak B., Christensen H., Emler S., Frey J., Kuhnert P. (2004). Phylogeny of the family *Pasteurellaceae* based on rpoB sequences. Int. J. Syst. Evol. Microbiol..

[32] Heuzenroeder M.W., Wong C.Y., Flower R.L. (1999). Distribution of two hemolytic toxin genes in clinical and environmental isolates of *Aeromonas* spp.: Correlation with virulence in a suckling mouse model. FEMS Microbiol. Lett..

[33] Kingombe C.I.B., Huys G., Tonolla M., Albert M.J., Swings J., Peduzzi R., Jemmi T. (1999). PCR detection, characterization, and distribution of virulence genes in *Aeromonas* spp.. Appl. Environ. Microbiol..

[34] Nawaz M., Khan S.A., Khan A.A., Sung K., Tran Q., Kerdahi K., Steele R. (2010). Detection and characterization of virulence genes and integrons in *Aeromonas veronii* isolated from catfish. Food Microbiol..

[35] Patel J.B., Cockerill F.R., Bradfoord P.A. (2015). CLSI M100-S25 performance standards for antimicrobial susceptibility testing; twenty-fifth informational supplement. Clin. Lab. Stand. Inst..

[36] Hedberg N., Stenson I., Pettersson M.N., Warshan D., Nguyen-Kim H., Tedengren M., Kautsky N. (2018). Antibiotic use in Vietnamese fish and lobster sea cage farms; implications for coral reefs and human health. Aquaculture.

[37] Hoa P.T.P., Managaki S., Nakada N., Takada H., Shimizu A., Anh D.H., Viet P.H., Suzuki S. (2011). Antibiotic contamination and occurrence of antibiotic-resistant bacteria in aquatic environments of northern Vietnam. Sci. Total Environ..

[38] Binh V.N., Dang N., Anh N.T.K., Thai P.K. (2018). Antibiotics in the aquatic environment of Vietnam: Sources, concentrations, risk and control strategy. Chemosphere.

[39] Do T.C.M.V., Nguyen D.Q., Nguyen T.D., Le P.H. (2020). Development and validation of a LC-MS/MS method for determination of multi-class antibiotic residues in aquaculture and river waters, and photocatalytic degradation of antibiotics by TiO_2_ nanomaterials. Catalysts.

[40] Krumperman P.H. (1983). Multiple antibiotic resistance indexing of *Escherichia coli* to identify high-risk sources of fecal contamination of foods. Appl. Environ. Microbiol..

[41] Van Huong N., Cuong T.H., Thu T.T.N., Lebailly P. (2017). Efficiency of Different Integrated Agriculture Aquaculture Systems in the Red River Delta of Vietnam. Fish. Aquac. J..

[42] Igbinosa I.H., Igumbor E.U., Aghdasi F., Tom M., Okoh A.I. (2012). Emerging *Aeromonas* species infections and their significance in public health. Sci. World J..

[43] Pal M. (2018). Is *Aeromonas hydrophila* a potential pathogen of food safety concern. J. Food Microbiol..

[44] Hoel S., Vadstein O., Jakobsen A.N. (2019). The significance of mesophilic *Aeromonas* spp. in minimally processed ready-to-eat seafood. Microorganisms.

[45] Parker J.L., Shaw J.G. (2011). *Aeromonas* spp. clinical microbiology and disease. J. Infect..

[46] Vila J., Marco F., Soler L., Chacon M., Figueras M.J. (2002). In vitro antimicrobial susceptibility of clinical isolates of *Aeromonas caviae, Aeromonas hydrophila* and *Aeromonas veronii* biotype sobria. J. Antimicrob. Chemothe..

[47] Li J., Ni X.D., Liu Y.J., Lu C.P. (2011). Detection of three virulence genes alt, ahp and aerA in *Aeromonas hydrophila* and their relationship with actual virulence to zebrafish. J. Appl. Microbiol..

[48] Jiravanichpaisal P., Roos S., Edsman L., Liu H., Söderhäll K. (2009). A highly virulent pathogen, *Aeromonas hydrophila*, from the freshwater crayfish *Pacifastacus leniusculus*. J. Invertebr. Pathol..

[49] Nicholson P., Mon-on N., Jaemwimol P., Tattiyapong P., Surachetpong W. (2020). Coinfection of tilapia lake virus and *Aeromonas hydrophila* synergistically increased mortality and worsened the disease severity in tilapia (*Oreochromis* spp.). Aquaculture.

[50] Xu X.J., Ferguson M.R., Popov V.L., Houston C.W., Peterson J.W., Chopra A.K. (1998). Role of a cytotoxic enterotoxin in *Aeromonas*-mediated infections: Development of transposon and isogenic mutants. Infect. Immun..

[51] El-Bahar H.M., Ali N.G., Aboyadak I.M., Khalil S.A.E.S., Ibrahim M.S. (2019). Virulence genes contributing to *Aeromonas hydrophila* pathogenicity in *Oreochromis niloticus*. Int. Microbiol..

[52] Oliveira S.T., Veneroni-Gouveia G., Costa M.M. (2012). Molecular characterization of virulence factors in *Aeromonas hydrophila* obtained from fish. Pesquisa Veterinária Brasileira.

[53] Ristow L.C., Welch R.A. (2016). Hemolysin of uropathogenic *Escherichia coli*: A cloak or a dagger?. Biochimica et Biophysica Acta (BBA)-Biomembranes.

[54] Tomás J.M. (2012). The main *Aeromonas* pathogenic factors. ISRN Microbiol..

[55] Hayati H.R., Hassan M., Ong B., Abdelhadi Y., Hidayahanum H.N., Sharifah R., Faten A.N., Kuttichantran S., Alsaid M. (2015). Virulence genes detection of *Aeromonas hydrophila* originated from diseased freshwater fishes. Adv. Environ. Biol..

[56] Chopra A.K., Houston C.W. (1999). Enterotoxins in *Aeromonas*-associated gastroenteritis. Microbes Infect..

[57] Rather M.A., Willayat M.M., Wani S.A., Hussain S.A., Shah S.A. (2019). Enterotoxin gene profile and molecular epidemiology of *Aeromonas* species from fish and diverse water sources. J. Appl. Microbiol..

[58] Abd El Tawab A.A., Maarouf A.A., El Hofy F.I., El Mougy E.E. (2017). Detection of some virulence genes in *A. hydrophila* and *A. caviae* isolated from fresh water fishes at Qalubia Governorate. Benha Vet. Med. J..

[59] Hoel S., Vadstein O., Jakobsen A.N. (2017). Species distribution and prevalence of putative virulence factors in mesophilic *Aeromonas* spp. isolated from fresh retail sushi. Front. Microbiol..

[60] Aoki T., Michel C., Alderman D. (1992). Present and future problems concerning the development of resistance in aquaculture. Chemotherapy in Aquaculture: From Theory to Reality.

[61] Akinbowale O.L., Peng H., Barton M. (2006). Antimicrobial resistance in bacteria isolated from aquaculture sources in Australia. J. Appl. Microbiol..

[62] WHO (2004). Joint FAO/OIE/WHO Expert Workshop on Non-Human Antimicrobial Usage and Antimicrobial Resistance: Scientific Assessment: Geneva, 1–5 December 2003 (No. WHO/CDS/CPE/ZFK/2004.7).

[63] Stratev D., Stoev S., Vashin I., Daskalov H. (2015). Some varieties of pathological changes in experimental infection of carps (*Cyprinus carpio*) with *Aeromonas hydrophila*. J. Aquac. Eng. Fish. Res..

[64] Fosse T., Giraud-Morin C., Madinier I. (2003). Phenotypes of beta-lactam resistance in the genus *Aeromonas*. Pathologie-Biologie.

[65] Borella L., Salogni C., Vitale N., Scali F., Moretti V.M., Pasquali P., Alborali G.L. (2020). Motile aeromonads from farmed and wild freshwater fish in northern Italy: An evaluation of antimicrobial activity and multidrug resistance during 2013 and 2016. Acta Vet. Scand..

[66] Dahanayake P.S., Hossain S., Wickramanayake M.V.K.S., Heo G.J. (2020). Prevalence of virulence and antimicrobial resistance genes in *Aeromonas* species isolated from marketed cockles (*Tegillarca granosa*) in Korea. Lett. Appl. Microbiol..

[67] Yuan K., Wang X., Chen X., Zhao Z., Fang L., Chen B., Jiang J., Luan T., Chen B. (2019). Occurrence of antibiotic resistance genes in extracellular and intracellular DNA from sediments collected from two types of aquaculture farms. Chemosphere.

[68] Ottaviani D., Santarelli S., Bacchiocchi S., Masini L., Ghittino C., Bacchiocchi I. (2006). Occurrence and characterization of *Aeromonas* spp. in mussels from the Adriatic Sea. Food Microbiol..

