# Supplementary material for: Prevalence, Virulence Gene Distribution and Alarming the Multidrug Resistance of Aeromonas hydrophila Associated with Disease Outbreaks in Freshwater Aquaculture
Source: Antibiotics (Basel). 2021 May 4;10(5):532. doi: 10.3390/antibiotics10050532 (PMC8147934; doi:10.3390/antibiotics10050532)
Supplement: Supplementary file 1 [file antibiotics-10-00532-s001.zip › antibiotics-1187108-supplementary.pdf]

Table S1. Biochemical characterization of *A. hydrophila* in the present study

Tests	*A. hydrophila* isolates in the present study			*A. hydrophila* ATCC 7966 (1)
	**Tilapia (n=187)**	**Carp (n=156)**	**Channel catfish (n=115)**	
Growth on RS medium (small, smooth, and yellow colonies	187	156	115	+
Cell morphology rods	187	156	115	+
Motility	187	156	115	+
Catalase	187	156	115	+
Cytochrome oxidase (OX)	187	156	115	+
Voges–Proskauer (VP)	155	136	97	+
Haemolysis of sheep RBCs	187	156	115	+
Sensitivity to 0/129 (150μg)	187	156	115	+
Indole production (IND)	148	129	97	+
Urease production (URE)	0	0	0	-
Citrate utilization (CIT)	162	139	93	+
Glucose fermentation	158	145	101	+
H_2S production	145	148	97	+
Arginine dihydrolase (ADH)	187	156	115	+
Lysine decarboxylase (LDC)	187	156	115	+
Ornithine decarboxylase (ODC)	0	0	0	-
Melibiose fermentation (MEL)	0	0	0	-
Amygdalin fermentation (AMY)	24	19	14	-
Arabinose fermentation (ARA)	173	138	101	+
Inositol fermentation (INO)	28	21	12	-
Mannitol fermentation (MAN)	154	146	93	+
Tryptophane deaminase (TDA)	0	0	0	-
Gelatin hydrolysis (GEL)	165	135	91	+

Table S2. The identities of gyrB and rpoB sequences from *A. hydrophila* in present study and those of *A. hydrophila* ATCC ATCC 7966

Isolates	gyrB (%)	rpoB (%)	Isolates	gyrB (%)	rpoB (%)	Isolates	gyrB (%)	rpoB (%)	Isolates	gyrB (%)	rpoB (%)
Til-LC01	99.2	98.8	Til-HD06	99.2	98.8	Carp-VP02	99.1	98.6	Carp-TB02	99.2	98.8
Til-LC02	99.1	98.6	Til-HD07	99.1	98.8	Carp-VP03	99.1	98.4	Carp-TB03	99.2	99.03
Til-LC03	99.1	98.8	Til-HD08	99.1	99.03	Carp-VP04	99.1	98.6	Carp-TB04	99.1	98.8
Til-LC04	99.1	98.8	Til-HY01	99.1	98.8	Carp-VP05	99.1	98.6	Carp-TB05	99.1	98.8
Til-SL01	99.1	98.8	Til-HY02	99.1	99.03	Carp-BG01	99.2	98.6	Carp-TB06	99.1	98.8
Til-SL02	99.1	99.03	Til-HY03	99.1	98.6	Carp-BG02	99.1	98.4	Chanfish-LC01	99.1	99.03
Til-SL03	99.1	98.8	Til-HY04	99.1	98.6	Carp-BG03	99.1	98.6	Chanfish-LC02	99.1	98.8
Til-SL04	99.2	99.03	Til-HB01	99.2	98.6	Carp-BG04	99.1	98.6	Chanfish-SL01	99.1	99.03
Til-SL05	99.2	98.6	Til-HB02	99.1	98.4	Carp-BG05	99.1	98.6	Chanfish-SL02	99.2	98.6
Til-SL06	99.2	98.6	Til-HB03	99.1	98.6	Carp-BG06	99.1	98.8	Chanfish-YB01	99.1	98.6
Til-YB01	99.2	98.6	Til-HB04	99.1	98.6	Carp-BG07	99.2	98.6	Chanfish-YB02	99.1	98.6
Til-YB02	99.2	98.4	Til-HB05	99.1	98.6	Carp-BN01	99.2	98.8	Chanfish-YB03	99.1	98.4
Til-YB03	99.1	98.6	Til-HB06	99.1	98.4	Carp-BN02	99.2	98.8	Chanfish-YB04	99.1	98.6
Til-YB04	99.1	98.6	Til-HN01	99.2	98.6	Carp-BN03	99.2	98.8	Chanfish-YB05	99.1	98.6
Til-YB05	99.1	98.6	Til-HN02	99.2	98.6	Carp-BN04	99.2	99.03	Chanfish-YB06	99.2	98.6
Til-YB06	99.1	98.8	Til-HN03	99.2	98.6	Carp-BN05	99.1	98.8	Chanfish-YB07	99.1	98.4
Til-YB07	99.1	98.6	Til-HN04	99.2	98.8	Carp-BN06	99.1	98.8	Chanfish-YB08	99.1	98.6
Til-TQ01	99.1	98.8	Til-HN05	99.2	98.6	Carp-QN01	99.1	98.8	Chanfish-YB09	99.1	98.6
Til-TQ02	99.2	98.8	Til-HN06	99.1	98.8	Carp-QN02	99.1	99.03	Chanfish-TQ01	99.1	98.6
Til-TQ03	99.2	98.8	Til-TH01	99.1	98.8	Carp-QN03	99.1	98.8	Chanfish-TQ02	99.1	98.8
Til-TQ04	99.2	99.03	Til-TH02	99.1	98.8	Carp-QN04	99.1	99.03	Chanfish-PT01	99.2	98.6
Til-TQ05	99.2	98.8	Til-TH03	99.1	99.03	Carp-QN05	99.2	98.6	Chanfish-PT02	99.2	98.8
Til-TQ06	99.2	99.03	Til-TH04	99.1	98.8	Carp-QN06	99.1	98.6	Chanfish-VP01	99.2	98.8
Til-TQ07	99.1	98.6	Til-TH05	99.1	99.03	Carp-HD01	99.1	98.6	Chanfish-VP02	99.2	98.8
Til-TQ08	99.1	98.6	Til-NA01	99.2	98.6	Carp-HD02	99.1	98.4	Chanfish-BG01	99.2	99.03
Til-PT01	99.1	98.6	Til-NA02	99.1	98.6	Carp-HD03	99.1	98.6	Chanfish-BG02	99.1	98.6
Til-PT02	99.1	98.4	Til-NA03	99.1	98.6	Carp-HD04	99.1	98.6	Chanfish-BN01	99.1	98.8
Til-PT03	99.1	98.6	Til-NA04	99.1	98.4	Carp-HD05	99.2	98.6	Chanfish-BN02	99.1	98.8
Til-PT04	99.1	98.6	Til-NA05	99.1	98.8	Carp-HD06	99.1	98.4	Chanfish-BN03	99.1	98.8
Til-PT05	99.2	98.6	Til-NA06	99.1	98.8	Carp-HY01	99.1	98.6	Chanfish-BN04	99.1	99.03
Til-VP01	99.2	98.6	Til-TB01	99.1	98.8	Carp-HY02	99.1	98.6	Chanfish-BN05	99.1	98.8
Til-VP02	99.2	98.6	Til-TB02	99.2	99.03	Carp-HY03	99.1	98.6	Chanfish-BN06	99.2	98.8
Til-VP03	99.2	98.6	Til-TB03	99.1	98.8	Carp-HY04	99.1	98.8	Chanfish-QN01	99.1	98.8
Til-VP04	99.1	98.4	Til-TB04	99.1	99.03	Carp-HB01	99.2	98.6	Chanfish-QN02	99.1	99.03
Til-VP05	99.1	98.6	Carp-LC01	99.1	98.6	Carp-HB02	99.2	98.8	Chanfish-HD01	99.1	98.8
Til-BG01	99.1	98.6	Carp-LC02	99.1	98.6	Carp-HB03	99.2	98.8	Chanfish-HD02	99.1	99.03
Til-BG02	99.1	98.6	Carp-LC03	99.1	98.6	Carp-HB04	99.2	98.8	Chanfish-HD03	99.1	98.6

Til-BG03	99.1	98.8	Carp-LC04	99.1	98.4	Carp-HB05	99.2	99.03	Chanfish-HD04	99.2	98.6
Til-BG04	99.2	98.6	Carp-LC05	99.2	98.6	Carp-HB06	99.1	98.8	Chanfish-HD05	99.1	98.6
Til-BG05	99.2	98.8	Carp-SL01	99.1	98.6	Carp-HB07	99.1	98.8	Chanfish-HD06	99.1	98.4
Til-BG06	99.2	98.8	Carp-SL02	99.2	98.6	Carp-HN01	99.1	98.8	Chanfish-HD07	99.1	98.6
Til-BN01	99.2	98.8	Carp-SL03	99.1	98.4	Carp-HN02	99.1	99.03	Chanfish-HY01	99.1	98.6
Til-BN02	99.2	99.03	Carp-SL04	99.1	98.6	Carp-HN03	99.1	98.8	Chanfish-HY02	99.1	98.6
Til-BN03	99.1	98.8	Carp-YB01	99.1	98.6	Carp-HN04	99.1	99.03	Chanfish-HB01	99.2	98.4
Til-BN04	99.1	99.03	Carp-YB02	99.1	98.6	Carp-HN05	99.2	98.6	Chanfish-HB02	99.2	98.6
Til-BN05	99.1	98.6	Carp-YB03	99.1	98.8	Carp-HN06	99.1	98.6	Chanfish-HN01	99.2	98.6
Til-BN06	99.1	98.6	Carp-YB04	99.2	98.6	Carp-HN07	99.1	98.6	Chanfish-HN02	99.2	98.6
Til-QN01	99.1	98.6	Carp-YB05	99.2	98.8	Carp-HN08	99.1	98.4	Chanfish-TH01	99.2	98.8
Til-QN02	99.1	98.4	Carp-YB06	99.2	98.8	Carp-TH01	99.1	98.6	Chanfish-TH02	99.1	98.6
Til-QN03	99.2	98.6	Carp-TQ01	99.2	98.8	Carp-TH02	99.1	98.6	Chanfish-NA01	99.1	98.8
Til-QN04	99.1	98.6	Carp-TQ02	99.2	99.03	Carp-TH03	99.2	98.6	Chanfish-NA02	99.1	98.8
Til-QN05	99.1	98.6	Carp-TQ03	99.1	98.8	Carp-TH04	99.1	98.4	Chanfish-TB01	99.1	98.8
Til-QN06	99.1	98.4	Carp-TQ04	99.1	98.8	Carp-TH05	99.1	98.6	Chanfish-TB02	99.1	99.03
Til-QN07	99.1	98.6	Carp-TQ05	99.1	98.8	Carp-NA01	99.1	98.6	Chanfish-TB03	99.1	98.6
Til-HD01	99.1	98.6	Carp-PT01	99.1	99.03	Carp-NA02	99.1	98.6	Chanfish-TB04	99.2	98.6
Til-HD02	99.2	98.6	Carp-PT02	99.1	98.8	Carp-NA03	99.1	98.8	Chanfish-TB05	99.1	98.4
Til-HD03	99.2	98.8	Carp-PT03	99.1	99.03	Carp-NA04	99.2	98.6	Chanfish-TB06	99.1	98.6
Til-HD04	99.2	98.6	Carp-PT04	99.2	98.6	Carp-NA05	99.2	98.8	Chanfish-TB07	99.1	98.6
Til-HD05	99.2	98.8	Carp-VP01	99.1	98.6	Carp-TB01	99.2	98.8	Chanfish-TB08	99.1	98.6

Table S3. Primers used for the detection of virulence genes in *A. hydrophila*

Gene	DNA sequence (5′→3′)	Product size (bp)	Tm(°C)	References
act	F: AGAAGGTGACCACCACCAAGAACA<br>R: AACTGACATCGGCCTTGAACTC	232	65	[33]
alt	F: TGACCCAGTCCTGGCACGGC<br>R: GGTGATCGATCACCACCAGC	442	64	[33]
ast	F: TCTCCATGCTTCCCTTCCACT<br>R: GTGTAGGGATTGAAGAAGCCG	331	63	[33]
aerA	F: CCTATGGCCTGAGCGAGAAG<br>R: CCAGTTCCAGTCCCACCACT	431	63	[34]
hlyA	F: GGCCGGTGGCCCGAAGATACGGG<br>R: GGCGGCGCCGGACGAGACGGG	597	62	[32]
